# Examining the Gender Disparities in Health Service Utilization Among Older Adults Managing Cognitive Impairments

**DOI:** 10.1093/geroni/igab046.1852

**Published:** 2021-12-17

**Authors:** Camille McKenzie, Patricia Findley

**Affiliations:** 1 Rutgers University--New Brunswick, New Jersey, United States; 2 Rutgers University--New Brunswick, Rutgers University--New Brunswick, New Jersey, United States

## Abstract

Comorbid conditions can complicate healthcare access, yet little is known about the impact of cognition on accessing those services. This study aims to examine the effect of gender on health service utilization among the aging population. Data from the National Health Interview (2019) Survey (n=3,058) were utilized to assess the impact of gender on the use of physician and dental services among older adults managing cognitive impairments. We estimated logistic regression models controlling for demographic, health/physical functioning, and psychological predictors. Findings indicated older male respondents managing cognitive impairments were less inclined to utilize both physician (p<.05) and dental (p<.01) health services on a yearly basis as compared to their female counterparts. Household income (aOR = 0.62 [-0.91, -0.06]; aOR = 0.34 [-1.25, -0.90]) and health insurance coverage (aOR = 0.18 [-2.31, -1.13]; aOR = 0.32 [-1.68, -0.62]) respectively were the strongest structural factors limiting access to both physician and dental services, with lower levels of educational attainment (aOR = 0.46 [-1.00, -0.55]) being an additional barrier for dental service utilization. Respondents with low self-rated health were inclined to utilize physician more than dental services (aOR = 2.31 [0.38, 1.29]; aOR = 0.73 [-0.50, -0.14]). It is imperative geriatric practitioners adopt a more integrative approach to health, especially among the aging population managing chronic disabilities, where additional community supports are warranted to promote and maintain physical and oral health.

